# Chemotherapy-Induced Neutropenia in Palestinian Patients with Solid Malignancies: Patient Characteristics, Severity Risk Factors, and Management Patterns

**DOI:** 10.1158/2767-9764.CRC-25-0770

**Published:** 2026-07-17

**Authors:** Natalie Khamashta, Ahmad Dalal, Mo’men Alashwas, Musab Hamdan, Fuad Al-Rimawi, Yousef Sahoury

**Affiliations:** 1 Faculty of Medicine, Al-Quds University, Jerusalem, Palestine.; 2 Department of Oncology, Palestine Medical Complex, Ramallah, Palestine.; 3 Chemistry Department, Faculty of Science and Technology, Al-Quds University, Jerusalem, Palestine.; 4 Pharmacology and Physiology Department, Faculty of Medicine, Al-Quds University, Jerusalem, Palestine.

## Abstract

Neutropenia is an adverse event of systemic chemotherapy with various complications that affect patient management, quality of life, and could be fatal. The study was conducted to evaluate chemotherapy-induced neutropenia (CIN) in a Palestinian cohort of patients with solid malignancies. This article examines patient characteristics, risk factors for neutropenia severity, and neutropenia management patterns. The study included a total of 171 adult patients diagnosed with solid malignancies who were on their first chemotherapy regimens with no history of neutropenia. Data were collected retrospectively from patients’ electronic medical records. Only patients who developed neutropenia during treatment were included in this study, and patients were classified into three groups of severity based on the absolute neutrophil count (ANC). Colon cancer and lung cancer were the most reported. Epirubicin, paclitaxel, cyclophosphamide, and doxorubicin were significantly associated with neutropenia severity, in addition to the number of chemotherapy cycles, stage at diagnosis, and certain comorbidities. Of the total patients, 32.5% reported substance use which was also significantly associated with severity. The majority of patients received primary granulocyte colony-stimulating factor (GCSF) prophylaxis. Sixty-nine percent of patients presented with febrile neutropenia. Hospitalization, antibiotics, antipyretics, and GCSF were utilized for management in most patients. Neutropenia severity was associated with the chemotherapy agents used, whereas the type of solid malignancy was not. Substance use represented a high burden among our cohort, and its association with severity could be further studied. Primary GCSF prophylaxis is critical, with patients not receiving it developing profound neutropenia (ANC <0.1 × 10^9^) at higher rates.

**Significance:**

CIN causes high utilization of medical resources and disrupts treatment schedules. Factors possibly associated with severity include stage at diagnosis, number of chemotherapy cycles, select agents, and comorbidities. GCSF prophylaxis possibly decreases profound neutropenia rates. Palestinian patients with cancer have a high burden of substance use, possibly reflecting unmet needs.

## Introduction

Chemotherapy-induced neutropenia (CIN) is a principal dose-limiting toxicity in systemic therapy for solid malignancies and the proximate driver of febrile neutropenia (FN), unplanned hospitalization, and early mortality (1). According to the Common Terminology Criteria for Adverse Events, neutropenia is graded by absolute neutrophil count (ANC) as follows: grade 1, 1.5 to 2 × 10^9^/L; grade 2, 1 to 1.5 × 10^9^/L; grade 3, 0.5 to 1 × 10^9^/L; and grade 4, <0.5 × 10^9^/L (2).

FN is a detrimental complication of CIN. Per joint American Society of Clinical Oncology (ASCO)/Infectious Diseases Society of America guidance, FN is defined as a single oral temperature ≥38.3°C, or a sustained ≥38°C for ≥1 hour, with ANC <0.5 × 10^9^/L or ANC expected to fall <0.5 × 10^9^/L within 48 hours. Within this framework, severe neutropenia is ANC <0.5 × 10^9^/L and profound neutropenia is ANC <0.1 × 10^9^/L (1).

FN incidence varies by regimen and host-level risk, generally 8% to 15% in modern series, with population-level estimates up to 50% in some studies (3–5). Despite advances in supportive care, FN still confers nontrivial short-term mortality, commonly 3% to 10% in high-resource settings; another study estimated a mortality rate of 5% to 11%, which can increase to 50% in certain high-risk populations (6, 7).

Severity and duration of neutropenia strongly influence outcomes. In a solid tumor series of 1,947 patients receiving intravenous chemotherapy, mortality per FN episode approached 15%, and ANC <0.1 × 10^9^/L independently predicted death, underscoring the disproportionate hazard of profound neutropenia (4).

According to the Palestinian Ministry of Health 5-year cancer report 2017 to 2021, cancer incidence is 119.2 per 100,000 in 2021, which is double that in 2010. Breast cancer is the most common (15.9% of all cancer types), followed by colorectal (14.7%) and lung (7.7%) cancers. This highlights the increasing demand on oncologic healthcare in Palestine and the potentially increasing rates of treatment complications, including neutropenia (8).

In this retrospective observational study of 171 adults with solid malignancies who developed CIN, we investigated severity, risk factors and associations, management approaches, and care utilization.

## Materials and Methods

### Ethical approval and informed consent

This study was conducted in accordance with the Declaration of Helsinki and in compliance with relevant laws and institutional guidelines. Ethical approval (reference number: 398/REC/2024, date: May 21, 2024) was obtained from the Institutional Review Board (IRB) and ethics committee at Al-Quds University prior to data collection. Informed consent was waived by the IRB and ethics committee because of the retrospective nature of the study. Data were retrospectively collected from three oncology centers in the West Bank: Beit Jala Hospital, Augusta Victoria Hospital, and Palestine Medical Complex between the years 2022 and 2025. Data were extracted directly from patient records while ensuring patient privacy and anonymity.

### Study population and sample size

Inclusion criteria: adult patients (>18 years) who were diagnosed with solid malignancies between 2022 and 2025 and on their first chemotherapy-based regimens. Only patients who have developed neutropenia (ANC <1 × 10^9^/L) at some point during their treatment journey were included, and they must have never received a diagnosis of neutropenia for any other reason before. All included patients reside in the West Bank or Jerusalem and have received cancer treatment in Beit Jala Hospital, Augusta Victoria Hospital, or Palestine Medical Complex. Male and female patients were both included in the study.

A predetermined number of 170 patients was selected. This number was chosen to adequately represent the Palestinian population. By the end of the study, 171 patients in total were included.

### Statistical analysis

Analyses were performed using SPSS v25 (RRID: SCR_002865). We conducted data cleaning and coding, handled missing responses appropriately, and calculated valid percentages. Demographical data regarding age and general characteristics were studied. Descriptive statistics summarized categorical variables with frequencies and percentages; continuous variables were summarized using measures of central tendency and variability.

Associations between neutropenia severity and demographic, lifestyle, and clinical factors were tested using *χ*^2^. Association strength was interpreted based on Cramér’s V as weak (<0.1), moderate (0.1–0.3), and strong (>0.3).

Spearman correlation coefficients assessed relationships between continuous variables, such as baseline neutrophil counts, number of chemotherapy cycles, and neutropenia severity. This nonparametric measure was chosen when normality assumptions were violated, as indicated by Kolmogorov–Smirnov and Shapiro–Wilk tests.

To compare absolute neutrophil drop between patients who received prophylactic granulocyte colony-stimulating factor (GCSF) and those who did not, we used Mann–Whitney U tests. Neutropenia severity was computed based on posttreatment ANC as follows:

- Group 1: 0.5 to 1 × 10^9^/L.
- Group 2: 0.1 to 0.5 × 10^9^/L.
- Group 3: <0.1 × 10^9^/L.

The neutrophil drop was calculated as the difference between pretreatment and posttreatment counts.

### Study limitations

The retrospective nature of the study limited the availability of some clinical variables such as the exact timing of neutropenia onset following chemotherapy and confirmed microbiological sources of infection. Additionally, it limited the ability to perform complicated multivariate analysis.

## Results

**Figure 1.**
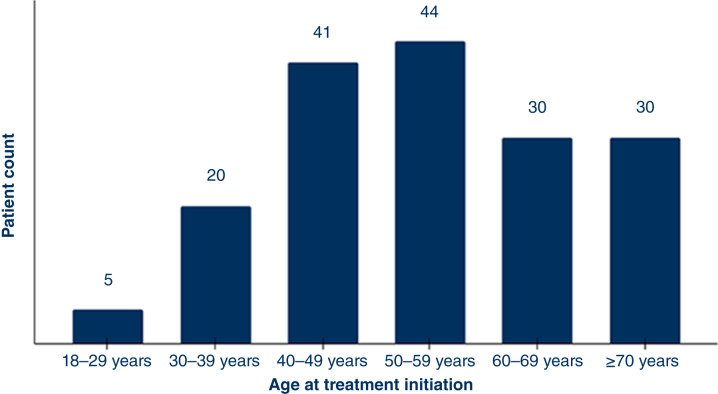
Age distribution of the patient population included in our cohort. *X* axis represents age at the time of treatment initiation. *Y* axis represents patient count.

Among 171 patients, 54.97% were male (*n* = 94) and 45.03% were female (*n* = 77). A total of 85.3% were older than 40 years; age distribution is shown in Fig. 1.

**Figure 2.**
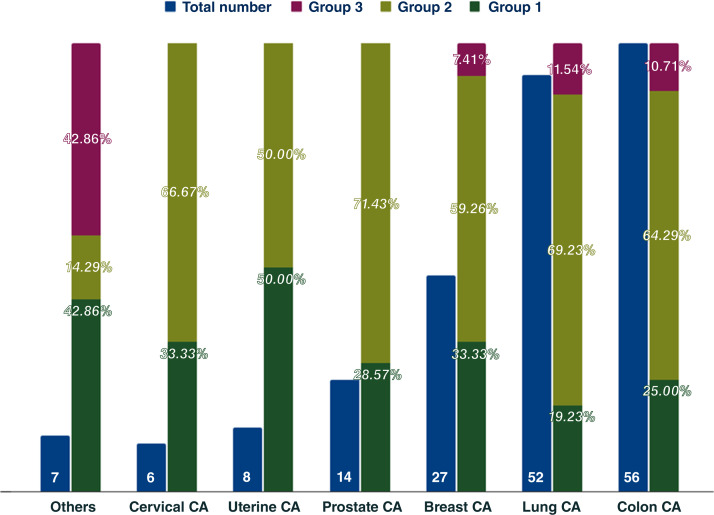
Different types of solid malignancies represented in our cohort. The left bar in each category represents the total number for each type of malignancy, and the right bar represents the percentage of each neutropenia severity group. Group 1 represents neutropenia with ANC 0.5–1 × 10^9^. Group 2 represents severe neutropenia with ANC 0.1–0.5 × 10^9^. Group 3 represents profound neutropenia with ANC less than 0.1 × 10^9^. CA, cancer.

Smoking was reported by 94 patients (55.3%), alcohol use by six patients (3.51%), and substance use by 55 patients (32.54%). Figure 2 shows the malignancy types in our cohort and their distribution among neutropenia groups.

At diagnosis, 84 patients (50.91%) were stage 3, 64 (38.79%) stage 2, and 17 (10.30%) stage 4. Overall, 52.44% presented with or later developed metastasis.

Most patients (83.04%) received radiotherapy, commonly three sessions (39.47%), four sessions (28.07%), or five sessions (14.04%). Additionally, 100 patients (58.82%) received hormonal therapy, and 23 reported using herbal therapy.

The single most used chemotherapeutic agent was oxaliplatin (71 patients, 41.52%), followed by fluorouracil, cyclophosphamide, carboplatin, cisplatin, docetaxel, and capecitabine (Table 1). A total of 95.32% patients received six or more cycles. A total of 97.66% patients received at least two chemotherapeutic agents.

**Table 1.** Utilization of chemotherapy drugs among patients (*n* = 171).

Drug	Count	%
Oxaliplatin	71	41.52%
Fluorouracil	57	33.33%
Cyclophosphamide	43	25.15%
Carboplatin	37	21.64%
Cisplatin	36	21.05%
Capecitabine	35	20.47%
Docetaxel	35	20.47%
Doxorubicin	31	18.13%
Paclitaxel	30	17.54%
Gemcitabine	28	16.37%
Epirubicin	18	10.53%
Methotrexate	5	2.92%
Others	3	1.75%

The mean baseline neutrophil count prior to neutropenia was 5.527 × 10^9^/L (median 5.600 × 10^9^/L; SD 1.537). The mean posttreatment count was 0.341 × 10^9^/L (median 0.300 × 10^9^/L; SD 0.230).

Most patients (76.61%) developed neutropenia following the third chemotherapy cycle; the average number of cycles prior to onset was 4.23 (SD 1.38).

**Figure 3.**
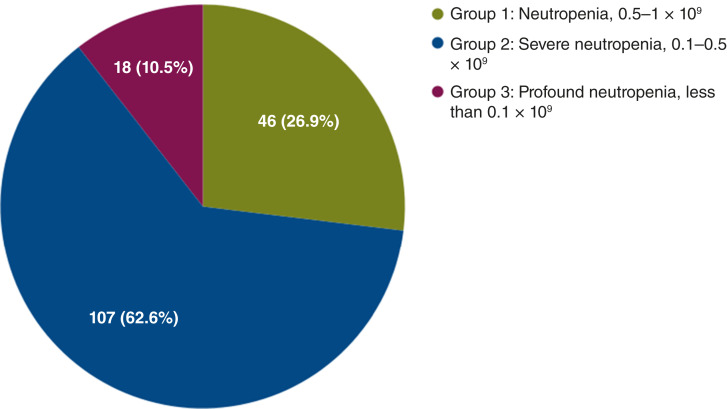
A pie chart that displays the distribution of our cohort into the three ASCO classification severity groups.

Patients were classified by severity of neutropenia according to the ASCO classification: group 1 (0.5–1 × 10^9^/L), group 2 (0.1–0.5 × 10^9^/L), and group 3 (<0.1 × 10^9^/L). Figure 3 shows the distribution.

### Risk factors

Analyzing demographic risk factors shows that gender was significantly associated with neutropenia severity (*χ*^2^ = 12.632, *P* = 0.002; Cramér’s V = 0.272, moderate). Age showed no significant association (*χ*^2^ = 4.053, *P* = 0.852; Cramér’s V = 0.109) and did not correlate with absolute neutrophil drop (Spearman −0.02, *P* = 0.794).

#### Lifestyle factors

Smoking showed a trend toward significance (*χ*^2^ = 5.538, *P* = 0.063; Cramér’s V = 0.180, moderate). Alcohol intake was not significantly associated with severity (*χ*^2^ = 0.767, *P* = 0.682), whereas substance use was (*χ*^2^ = 16.438, *P* < 0.001; Cramér’s V = 0.312, strong).

#### Cancer-related factors

Stage at diagnosis was significantly associated with neutropenia severity (*χ*^2^ = 21.322, *P* < 0.001; Cramér’s V = 0.254), unlike cancer type and metastasis status.

#### Therapy-related factors

Significant associations with severity were observed for epirubicin (*χ*^2^ = 9.150, *P* = 0.010), paclitaxel (10.490, *P* = 0.005), cyclophosphamide (8.716, *P* = 0.013), and doxorubicin (9.732, *P* = 0.008). The number of cycles prior to neutropenia onset correlated positively with severity (*r* = 0.175, *P* = 0.022). Radiotherapy showed no significant association (*χ*^2^ = 8.491, *P* = 0.075). Hormonal therapy was significantly associated with severity (*χ*^2^ = 30.686, *P* < 0.001; Cramér’s V = 0.3). Herbal therapy approached significance (*χ*^2^ = 9.366, *P* = 0.053; Cramér’s V = 0.166, moderate).

#### Comorbidities

Hypertension affected 67.84% (116 patients) and was significantly associated with severity (*χ*^2^ = 7.093, *P* = 0.029; Cramér’s V = 0.204). Diabetes mellitus was present in 37.43% (64 patients). Other conditions included obstructive lung disease (39 patients, 22.8%), gout, hyperlipidemia, heart failure, and hyperthyroidism. One patient had tuberculosis; no human immunodeficiency virus (HIV) cases were reported. Obstructive lung disease had a significant association with severity (*χ*^2^ = 8.185, *P* = 0.017).

#### Baseline neutrophils

Baseline neutrophil count showed no significant association with neutropenia severity or posttreatment neutrophil count (*P* = 0.424 and *P* = 0.641).

### Management of neutropenia

Neutropenia may be asymptomatic but often presents with infectious or systemic signs. Fever was most common (90.7%), closely followed by abdominal pain (89%) and painful urination and shortness of breath (each 83.1%). Other symptoms included inflammation around cuts and bruises, oral candidiasis, and vaginal discharge. Less frequent symptoms included severe constipation, bloody diarrhea, rash, migraine, and severe urinary tract infections.

#### Prophylaxis

Among the cohort, 142 patients (83.04%) received prophylactic GCSF; 29 did not. Prophylactic GCSF was significantly associated with severity (*χ*^2^ = 31.501, *P* < 0.001; Cramér’s V = 0.429, strong). Patients receiving prophylactic GCSF had a mean absolute neutrophil drop of 4.26 × 10^9^/L (SD 1.91), compared with 5.37 × 10^9^/L (SD 1.43) without GCSF. The Mann–Whitney U test was 1,428 (*P* = 0.009), indicating a significant difference in absolute drop between groups.

#### Febrile neutropenia

A total of 118 patients (69%) presented with FN. Of those, 82.2% (97) required hospitalization, 100% (118) received antibiotics and antipyretics, and 22% (26) received antifungals. Among patients with FN, 89% (105) had received prophylactic GCSF, whereas 11% (13) had not. Profound neutropenia occurred in 9.52% of those receiving prophylactic GCSF compared with 61.54% of those who did not. Overall, 15.3% (18) of patients with FN had profound neutropenia.

#### General management

Antibiotics and antipyretics were administered to 97% and 93.3% of patients, respectively. Hospitalization occurred in 84.2%. GCSF was administered to 82.6%. Antifungals were used in 20% (33). Transfusions were performed in 15.7% (27). Dose adjustments of causative agents occurred in 46.5% (80). Treatment cessation and cycle rescheduling were common approaches as well.

Common antibiotic regimens included piperacillin/tazobactam (Tazocin) plus amikacin (21.3%), meropenem plus amikacin (14.4%), and ceftriaxone plus amikacin (13.8%), among others.

## Discussion

Multiple risk factors contribute to CIN. Having one or more comorbidities has been associated with higher CIN incidence (9). A large study identified multiple comorbidities potentially associated with FN, including chronic obstructive pulmonary disease, congestive heart failure, renal disease, and autoimmune conditions, with HIV infection, liver disease, peptic ulcers, and thyroid disorders also positively associated with grade 3 to 4 neutropenia (10). In our cohort, hypertension and diabetes were the most prevalent comorbidities; only hypertension and obstructive lung disease showed significant associations with severity. The latter may reflect increased respiratory infection risk due to impaired respiratory protective mechanisms, altered microbiology, and inhaled corticosteroid use (11).

The relationship between gender and CIN is mixed. A multicenter study of 1,170 patients found that females were more likely to experience CIN and to develop higher-grade neutropenia (12). We observed a statistically significant moderate association between male gender and neutropenia severity, though the relationship is not necessarily linear and may be influenced by the higher male representation in our cohort (54.97%).

Age is often associated with neutropenia risk (13); older patients experience higher rates of complications, hospitalizations, and life-threatening infections (14, 15). Our cohort showed no association, likely reflecting a younger case mix, chemotherapy dose adjustments, and avoidance of multiagent regimens in older adults.

Lower baseline blood counts, including neutrophils, and low first-cycle nadirs consistently predict CIN risk (16). In our cohort, baseline neutrophil counts were not correlated with severity, suggesting limited predictive value.

Smoking has shown variable associations. One study of 1,242 patients with solid malignancies found that neutropenia was positively associated with smoking overall (9). However, several studies in gemcitabine-treated patients reported lower neutropenia rates among smokers, possibly due to higher myeloid progenitor proliferation (17, 18). In our study, smoking status showed a moderate association with severity that did not reach significance, and we found no evidence of a protective effect.

Substance use, mostly by obtaining controlled prescription-only medications through illegal channels, was significantly and strongly associated with neutropenia severity. With 55 patients reporting substance use, this finding indicates a substantial burden and highlights the need for further research into its role in CIN development and severity. This could also hint at unmet palliative needs for patients with cancer and the role of those agents in patient care.

Colorectal cancer was most prevalent in our cohort, followed closely by lung cancer. In the literature, commonly implicated tumors include breast, lung, colorectal, ovarian, and esophageal cancers; however, cancer type does not seem to influence neutropenia risk (19, 20, 21).

Advanced stage at diagnosis and metastasis have been linked to increased neutropenia risk. In breast cancer, advanced stage has been associated with more severe neutropenia; in lung cancer, advanced stage and the number of metastatic sites predicted severe neutropenia (22, 23). In elderly patients with various malignancies, more advanced stages at diagnosis significantly increased FN risk (24). In our population, metastasis was not significantly associated with severity, whereas stage at diagnosis showed a statistically significant moderate association.

Anticancer agents differ in cytotoxic potential and neutropenia risk. Alkylating agents, plant-derived alkaloids, antineoplastic antibiotics, and platinum agents are frequently implicated; these agents are also associated with infection once neutropenia occurs. Most protein kinase agents and immune checkpoint inhibitors are not prominently associated with neutropenia or its complications (25). In lung cancer, gemcitabine and taxanes confer higher risk, and cumulative numbers of agents add risk (23). In our cohort, epirubicin, paclitaxel, cyclophosphamide, and doxorubicin were significantly associated with neutropenia severity, reinforcing the influence of specific agents over primary tumor type.

Increasing numbers of chemotherapy cycles have been associated with higher risk and severity of neutropenia. In non–small cell lung cancer (NSCLC), >4 cycles significantly increased severe neutropenia risk (26). In breast cancer, greater numbers of planned cycles predicted grade 4 neutropenia (10). Our data align; we found a positive correlation between cycle count and severity, and most patients developed neutropenia after three cycles (mean 4.23).

Radiotherapy increases neutropenia risk, especially with concurrent chemotherapy and greater marrow irradiation (27). We found no significant association with severity in our cohort.

The role of herbal therapy remains unclear. One study found that concurrent herbal medicine increased CIN risk in advanced NSCLC, whereas another showed no effect (26, 28). Our findings showed a moderate association with severity and a trend toward significance but do not support a clear protective or risk modifying role.

Hormonal therapy does not clearly influence neutropenia risk in breast cancer, though hormone receptor status may be relevant. One study associated FN with estrogen receptor–negative disease and hormone receptor–negative/HER2-positive cancers (29). In prostate cancer, hormonal therapy has been associated with increased neutropenia risk, amplified when docetaxel is used (30–33). In our population, hormonal therapy was significantly associated with severity, potentially reflecting patient characteristics or concurrent treatments.

CIN frequently leads to FN, antibiotic use, hospitalization, and elevated short-term mortality. It also triggers dose reductions and delays in chemotherapy, with potential for worse outcomes (34).

Our cohort showed heavy care utilization: high rates of admission (84.2%), near-universal antibiotics (97%) and antipyretics (93.3%), frequent GCSF use (82.6%), transfusions in 15.7%, and medication adjustments in 46.5%, with common treatment cessations and cycle rescheduling.

GCSF reduces neutropenia complications in patients receiving chemotherapy (34). GCSF is a hematopoietic CSF that stimulates neutrophil precursor cells (35). Development of recombinant GCSF improved after myelosuppressive recovery and enabled administration of more potent chemotherapies with potential outcome benefits (35).

Current management guidelines for CIN aim to reduce FN risk, limit inpatient utilization, and improve overall survival by recommending GCSF as primary prophylaxis (35). The decision to use primary prophylaxis depends on the risk of FN associated with the regimen and on patient factors such as older age, female gender, poor performance status, comorbidities, nutritional status, history of FN, and recent surgery or open wounds (34).

In our cohort, 83.04% received prophylactic GCSF. We found a strong, significant association between prophylactic GCSF and severity; the average ANC drop was 1.11 × 10^9^/L lower in patients who received prophylaxis, supporting its use to attenuate severity and potentially reduce complications.

Chemotherapy regimens are classified by FN risk: high (≥20%), intermediate (10%–20%), and low (<10%; refs. 34, 36). The National Comprehensive Cancer Network and the European Organisation for Research and Treatment of Cancer list chemotherapy regimens and their risk of causing FN (34, 37, 38). Guidelines recommend initiation of primary GCSF prophylaxis with the first chemotherapy cycle and throughout treatment for high-risk regimens; for intermediate-risk regimens, prophylaxis is recommended for patients with at least one risk factor; low-risk regimens should not receive primary prophylaxis. GCSF use as primary prophylaxis reduces FN incidence, chemotherapy delays, and FN-related hospitalizations (34, 36).

GCSF is also used as secondary prophylaxis, initiated after the onset of CIN, in patients who did not receive primary prophylaxis but experienced FN or dose-limiting neutropenic events, and is continued throughout treatment (34).

FN management requires immediate hospitalization and initiation of intravenous empiric broad-spectrum antibiotics within 1 hour of admission, ideally after obtaining blood cultures, and continued until either targeted therapy is indicated or ANC and fever recover (5, 7, 34). FN may necessitate dose reductions, delays of ≥7 days, discontinuation, or regimen switching, all of which may compromise outcomes (7).

Gastrointestinal infections are the most common FN presentation, often due to translocation of gut bacteria after chemotherapy-induced mucosal injury. Signs of infection may be subtle, and fever may be the only symptom due to neutropenic patients having blunted inflammatory responses; therefore, any patient with neutropenic cancer with fever should be presumed to have FN until proven otherwise (5). Infections are often bacterial, whereas viral and fungal infections occur less frequently (5).

In our study, fever occurred in 90.7% of neutropenic patients. Abdominal pain, dysuria, and dyspnea were also frequent. Overall, 69% developed FN, likely an underestimate because it excludes patients whose ANC was expected to fall <500/μL within 48 hours. About 15% of patients with FN had profound neutropenia. More than 80% of patients with FN required hospitalization; all received antibiotics and antipyretics, and 22% received antifungals. Common antibiotic regimens included agents such as piperacillin/tazobactam, amikacin, meropenem, and ceftriaxone.

Among patients with FN, 90% had received primary prophylactic GCSF. Profound neutropenia developed in ∼10% receiving prophylaxis compared with ∼62% without, reinforcing the value of preventive therapy.

### Conclusion

Neutropenia remains one of the most important adverse events in patients receiving chemotherapy for solid tumors. It disrupts treatment schedules, leads to dose reductions and regimen changes, results in serious complications, including life-threatening infections, and frequently necessitates hospitalization and antibiotics. In our cohort, factors associated with greater severity included hypertension, obstructive lung disease, hormonal therapy, larger numbers of chemotherapy cycles, and specific agents such as epirubicin, doxorubicin, paclitaxel, and cyclophosphamide. The significant association between severity and substance use is notable and warrants further investigation. Prophylactic GCSF use seems critical, potentially preventing profound neutropenia and reducing absolute neutrophil decline during chemotherapy.

## Data Availability

The data generated in this study are not publicly available due to patient privacy, but the corresponding author can be contacted with reasonable requests to provide deidentified data.
